# Five-factor model personality traits in opioid dependence

**DOI:** 10.1186/1471-244X-7-37

**Published:** 2007-08-06

**Authors:** Hege Kornør, Hilmar Nordvik

**Affiliations:** 1 Norwegian Knowledge Centre for the Health Services, PO Box 7004 St. Olavsplass, 0130 Oslo, Norway; 2 Norwegian University of Science and Technology, Department of Psychology, 7491 Trondheim, Norway

## Abstract

**Background:**

Personality traits may form a part of the aetiology of opioid dependence. For instance, opioid dependence may result from self-medication in emotionally unstable individuals, or from experimenting with drugs in sensation seekers. The five factor model (FFM) has obtained a central position in contemporary personality trait theory. The five factors are: Neuroticism, Extraversion, Openness to Experience, Agreeableness and Conscientiousness. Few studies have examined whether there is a distinct personality pattern associated with opioid dependence.

**Methods:**

We compared FFM personality traits in 65 opioid dependent persons (mean age 27 years, 34% females) in outpatient counselling after a minimum of 5 weeks in buprenorphine replacement therapy, with those in a non-clinical, age- and sex-matched sample selected from a national database. Personality traits were assessed by a Norwegian version of the Revised NEO Personality Inventory (NEO PI-R), a 240-item self-report questionnaire. Cohen's d effect sizes were calculated for the differences in personality trait scores.

**Results:**

The opioid-dependent sample scored higher on Neuroticism, lower on Extraversion and lower on Conscientiousness (d = -1.7, 1.2 and 1.7, respectively) than the controls. Effects sizes were small for the difference between the groups in Openness to experience scores and Agreeableness scores.

**Conclusion:**

We found differences of medium and large effect sizes between the opioid dependent group and the matched comparison group, suggesting that the personality traits of people with opioid dependence are in fact different from those of non-clinical peers.

## Background

Opioid dependence is a severe condition associated with substantial psychological, social and medical impairment, as well as poor treatment outcomes. The aetiology of opioid dependence is not quite established. We believe a number of aspects are involved, including biological, psychological and socioeconomic factors [1,2].

According to the self-medication hypothesis [3-5], emotionally unstable individuals may experience that their psychological distress is alleviated when they use opioids. In that respect, using opioids can be seen as a response in a negative reinforcement process [6,7] – it removes an aversive stimulus (psychological distress), reinforcing the response (increased tendency to use opioids).

Opioid use has also been associated with sensation seeking and engagement in risk behaviours [8-10]. Zuckerman views sensation seeking as a personality trait with biological foundations, making some people more inclined to engage in risk behaviours than others [11].

The five-factor model (FFM) [12,13] of personality is a conceptualisation of personality comprising behavioural, emotional and cognitive patterns. These patterns are thought of as enduring dispositions which have proved to be stable from the age of 30 [14,15]. Also, the FFM has been reproduced in a number of culturally different countries [16-18], indicating a universally valid structure. The FFM has a hierarchical structure; each of the five domains Neuroticism, Extraversion, Openness to experience, Agreeableness and Conscientiousness is defined by six subdomains, or facets.

Studies of relationships between FFM dimensions and mental health indicate that people with psychiatric disorders have distinct personality patterns [19-22]. One meta-analysis found a general pattern of high Neuroticism, low Conscientiousness, low Agreeableness and low Extraversion in people with clinical symptoms [20]. Another meta-analysis identified high Neuroticism and low Agreeableness as underlying dimensions of most personality disorders [21]. People with various substance use disorders also seem to have a common personality profile: high Neuroticism, low Conscientiousness and low Agreeableness [23-29].

Two US studies have examined FFM personality traits in people with opioid dependence. Personality patterns were consistent with those of people with psychiatric and of people with substance use disorders, i.e. high Neuroticism, low Conscientiousness and low Agreeableness [30,31]. At the subdomain level, the largest deviations from norm scores were seen in Neuroticism facets Depression and Vulnerability, Agreeableness facets Trust and Straightforwardness, and Conscientiousness facets Competence, Dutifulness, Achievement Striving and Self-Discipline.

To these authors' knowledge there are no studies on the relationships between FFM personality traits and opioid dependence conducted outside the USA. Studies from other countries are needed to supplement US studies and the understanding of the origins and consequences of dependent opioid use. The aim of this study was to examine whether there is a distinct personality pattern associated with opioid dependence in young Norwegian adults, when compared with an age- and sex-matched non-clinical comparison group.

## Methods

### Opioid dependent sample

The opioid dependent sample was 65 participants in a feasibility trial of short-term buprenorphine replacement therapy in 2002–2003 [32,33]. Inclusion criteria in the feasibility trial were: age ≥ 22 years, opioid dependence diagnosed with the Composite International Diagnostic Interview [34], and enrolled in one of five specific outpatient clinics in South Eastern Norway. Persons with severe medical or psychiatric conditions or who had a prison sentence pending were excluded because they would be unable to adhere to the study protocol.

The sample's mean age was 26.8 years (SD 3.4; range 22–39), and 34% and 66% were women and men, respectively. Seven participants (11%) had more than 12 years' education and 18 (28%) were living with a partner. Thirty-nine participants (60%) had a lifetime mood disorder, 46 (70%) had an anxiety disorder and 52 (80%) had a personality disorder. Twenty-eight participants (43%) had spent more than 14 consecutive days in prison or custody. A majority of participants had used a number of illicit substances the last 30 days prior to intake to the feasibility trial (Table 1).

**Table 1:** Illicit substance use last 30 days prior to intake to trial for opioid dependent sample

	n (%)
Opioids	61 (94)
Sedatives	54 (83)
Cannabis	45 (69)
Amphetamines	20 (31)
Heavy drinking	10 (15)
Poly-substance use	51 (78)
Injecting use	51 (78)

A minimum of 5 weeks after buprenorphine induction, when assumed to have achieved a stable state, patients were requested to complete the NEO-PI-R at the clinics. Instructions were given both verbally and in writing, and clinic staff was available for answering any questions regarding the inventory. Approvals were granted from the Regional Committee for Medical Research Ethics, Norwegian Medicines Agency and the Data Inspectorate. All participants were informed both orally and in writing about the study, and signed informed consent forms.

### Matched comparison group

For each opioid dependent participant a comparison person with matching age and sex was randomly drawn from a national data base containing scores of 1153 individuals representing a wide range of the general Norwegian population. Individuals with known psychiatric disorder were removed from the database. Data from the comparison group were collected consecutively in various settings over a number of years (1998–2001). Thirty-two (49%) were graduate students and 20 (31%) were professionals. For the remaining comparison group we only know that 10 (15%) were participants in studies of physical activity or monozygotic twins.

### Analyses

The Norwegian version of the NEO-PI-R [35] was used for FFM personality trait assessments. Each individual's T-scores (mean = 50, SD = 10) were calculated on the basis of national combined norms.

Analyses were conducted using the statistical package SPSS for Windows, version 11.0 [36]. Cohen's *d *effect sizes [37] were calculated for the differences in T-scores between opioid dependent subjects and controls. Statistical power calculations showed that the sample size allowed the detection of a difference of a medium effect size (*d *≥ 0.50) at the 0.01-level with power > 0.8.

## Results

The non-clinical comparison group did not have any mean scores that deviated more than 2 points from the general norm mean (Table 2).

**Table 2:** Domain and facet level T-scores for opioid dependent sample and comparison sample

	Opioid dependent sample (N = 65)		Comparison sample (N = 65)			
	
	Mean	SD	Mean	SD	*d*	95% confidence interval
**DOMAIN LEVEL**						

NEUROTICISM *	64	8.0	49	8.9	1.74	1.3–2.14
EXTRAVERSION	41	8.4	51	8.9	1.17	0.80–1.54
OPENNESS TO EXPERIENCE	48	9.5	52	12.4	0.35	0.00–.69
AGREEABLENESS	46	8.3	51	11.0	0.49	0.14–.83
CONSCIENTIOUSNESS	36	8.1	50	9.3	1.67	1.27–2.07

**Neuroticism facets**						

Anxiety*	61	8.4	49	9.0	1.38	1.00–1.76
Angry hostility*	58	9.4	49	10.4	0.91	0.56–1.27
Depression*	65	7.0	49	8.9	2.02	1.59–2.44
Self-consciousness*	62	8.7	50	9.3	1.32	0.94–1.69
Impulsiveness*	54	8.3	52	10.1	0.29	0.00–0.63
Vulnerability*	63	9.6	49	8.6	1.54	1.15–-1.93

**Extraversion facets**						

Warmth	41	9.6	50	11.1	0.87	0.51–1.23
Gregariousness	41	10.6	50	10.4	0.89	0.52–1.25
Assertiveness	42	8.0	49	8.9	0.94	0.58–1.30
Activity	43	9.0	50	8.4	0.72	0.37–1.08
Excitement seeking*	54	7.9	52	10.0	-0.28	0.00–0.62
Positive emotions	40	7.7	53	9.0	1.57	1.17–1.96

**Openness to Experience facets**						

Fantasy	48	8.9	53	10.7	0.48	0.13–0.83
Aesthetics*	51	9.7	50	12.1	-0.11	0.00–0.44
Feelings	49	9.2	53	11.4	0.36	0.01–0.71
Actions*	49	8.2	48	10.0	-0.08	0.00–0.40
Ideas	47	10.6	52	11.7	0.45	0.10–0.79
Values	47	7.7	52	11.4	0.51	0.16–0.86

**Agreeableness facets**						

Trust	39	11.3	52	11.2	1.11	0.74–1.48
Straightforwardness	45	10.1	50	9.9	0.53	0.18–0.88
Altruism	47	9.3	50	12.0	0.31	0.04–0.65
Compliance	46	9.4	49	10.2	0.21	0.14–0.55
Modesty*	54	8.3	50	10.7	-0.47	0.15–0.81
Tender-mindedness*	53	8.5	52	12.1	-0.01	0.00–0.07

**Conscientiousness facets**						

Competence	35	10.9	52	10.1	1.53	1.14–1.92
Order	44	7.0	50	10.5	0.68	0.32–1.03
Dutifulness	38	8.5	51	9.4	1.47	1.08–1.86
Achievement striving	42	11.3	51	9.1	0.94	0.57–1.30
Self-discipline	38	7.8	50	9.6	1.36	0.97–1.74
Deliberation	39	7.5	48	10.8	0.97	0.61–1.34

* Negative *d *values

In the opioid dependent sample there were several deviations with medium (*d *≥ 0.50) or large (*d *≥ 0.80) effect sizes from the comparison group mean scores (Table 2):

• higher scores on Neuroticism and facets Anxiety, Angry Hostility, Depression, Self-Consciousness and Vulnerability

• lower scores on Conscientiousness and all facets

• lower scores on Extraversion and facets Warmth, Gregariousness, Assertiveness, Activity and Positive Emotions.

Moreover, the opioid dependent sample had lower scores on Agreeableness facets Trust and Straightforwardness. There were no medium or large effect sizes for any of the differences in Openness to Experience facets, except for Openness to Values (*d *= 0.51).

Almost half the opioid dependent sample (29; 45%) scored above the comparison sample's 95^th ^percentile on Neuroticism (T-score ≥ 63.95). Corresponding frequencies of extreme scorers (below the 5^th ^percentile) were 26%, 19%, 3% and 2% for Conscientiousness (T-score ≤ 29.93), Extraversion (T-score ≤ 33.11), Openness to Experience (T-score ≤ 31.18) and Agreeableness (T-score ≤ 28.44), respectively. When the 75^th ^and 25^th ^percentiles were used as cut-offs for extreme scorers, the frequencies were 88% for Neuroticism (T-score ≥ 55.67), 83% for Conscientiousness (T-score ≤ 45.12), 72% for Extraversion (T-score ≤ 46.99), 43% for Agreeableness (T-score ≤ 44.02) and 29% for Openness to Experience (T-score ≤ 42.05).

## Discussion

The Norwegian opioid dependent sample resembled the US ones [30,31] in terms of high Neuroticism, low Conscientiousness and average Openness to Experience. There were also dissimilarities between Norwegian and US findings: low Extraversion in Norwegian opioid users and no difference in Agreeableness between the opioid dependent and the comparison group.

Both this and the US studies of opioid dependent samples confirmed other research observations of high Neuroticism and low Conscientiousness in substance use disorders, across nationalities, sample age (US samples were older than the Norwegian one) and type of substances used (US participants were frequently co-dependent on cocaine). High Neuroticism in people with substance dependence can be seen as consistent with the self-medication hypothesis: people use and become dependent on opioids because they are emotionally unstable. However, we do not yet know the direction of causality, or whether one causes the other at all. Low Conscientiousness may be a common denominator for opioid use, risk behaviours and sensation seeking. Opioid users appear to share the low levels of Conscientiousness with people with risky health behaviours [30,38-41], and risk behaviours have been associated with sensation seeking [11].

The lack of differences between the two groups in Openness to Experience was also supportive of earlier work, suggesting that a person's degree of conventionality and adherence to traditions is unrelated to opioid dependence.

It is difficult to explain the low Extraversion in Norwegian opioid users. One approach is to see the high prevalence of psychiatric disorders in this sample in relation to previous studies showing strong associations between pure mood, anxiety or psychotic disorders and low Extraversion [20]. More knowledge about the mental health state in the US opioid dependent samples would of course shed light to such an approach.

Despite a lacking relationship between opioid dependence and Agreeableness at the domain level we found deviations that were consistent with US findings at the facet level. People with opioid dependence seem to be less trusting and less straightforward than the norm in both countries. Further, we calculated Cohen's *d *for Agreeableness in one of the US samples [31], which was 0.7, i.e. medium effect size. The correspondent Norwegian *d *was 0.49, i.e. approaching medium effect size. The apparent deviation in the Norwegian and US findings regarding Agreeableness may be a statistical artefact related to sample size. Further, while high Neuroticism and low Conscientiousness are consistent elements in substance use disorder personality profiles across studies [23,25-29], there are examples of studies where low Agreeableness failed to emerge [24,26,28], indicating a weaker association.

A limitation of this study is that we do not know to what extent participants were under the influence of illicit drugs when completing the personality inventory. In Carter and colleagues' study, all participants tested positive for illicit opioids or other drugs at the first administration of the NEO-PI-R, and 75% were using illicit substances during the second administration [31]. We did not administer the NEO-PI-R until the sixth week of buprenorphine replacement therapy, when patients were assumed to have achieved stability with regard to substance use. Data from the feasibility trial show that this assumption was rather reasonable. Assessments 3 months after the first buprenorphine dose showed that substance use was modest and significantly reduced since inclusion assessments [32].

There is also a possibility that both buprenorphine replacement therapy and counselling may have influenced the opioid dependent participants' personality trait scores. In that case we would expect even higher Neuroticism and lower Conscientiousness at intake to the feasibility trial and even more homogeneity in terms of high frequencies of extreme scores. Piedmont and colleagues found persisting changes in Neuroticism, Conscientiousness and Agreeableness in a sample with substance use disorders after a 6-week counselling programme [42].

Further, our study design can only identify associations with unknown causal directions. On one hand, the opioid dependent sample's distinct personality profile could be understood as part of the aetiology of opioid dependence. Several investigations have documented that personality traits are remarkably stable [14,15,43], that they have a significant hereditary component [44], and that they have behavioural implications, i.e., they influence behavior in any situation and they contribute to decisions on which situations individuals are motivated to enter and participate in [45]. On the other hand, the opioid-dependent sample's personality profile could be explained by a shared, distinctive lifestyle associated with long-term substance use. There is evidence suggesting that personality traits are less stable in younger adults than older adults [46], and thus more susceptible to external influences.

The five-factor model of personality has obtained a central position in contemporary personality trait theory. The impact of group personality profiles have been examined in several fields, including occupational psychology and mental health. Merely describing the personality characteristics of individuals with opioid dependence is not enough. We need to know more about how personality traits influence prognosis. It could also be useful to model treatment programmes using knowledge of the group's typical personality profile, and evaluating the effectiveness of such programmes compared to standard treatment.

## Conclusion

Patients with opioid dependence were more emotionally unstable, more introverted and less structured than the non-clinical controls. These findings may represent risk factors for opioid dependence, but may also be results of the lifestyles of illicit substance users. Nevertheless, the distinct personality profiles of opioid dependent patients may have implications for choice of therapeutic approach.

## Competing interests

The author(s) declare that they have no competing interests.

## Authors' contributions

HK administered personality trait assessments among the opioid dependent participants, performed the statistical analyses and drafted the manuscript. HN was responsible for data collection to the national database, and contributed to the statistical analyses and to drafting the manuscript. Both authors participated in the design of the study and read and approved the final manuscript.

## Pre-publication history

The pre-publication history for this paper can be accessed here:


